# Diagnostic Approach to Pediatric Autoimmune Neuropsychiatric Disorders Associated With Streptococcal Infections (PANDAS): A Narrative Review of Literature Data

**DOI:** 10.3389/fped.2021.746639

**Published:** 2021-10-27

**Authors:** Adriana Prato, Mariangela Gulisano, Miriam Scerbo, Rita Barone, Carmelo M. Vicario, Renata Rizzo

**Affiliations:** ^1^Department of Cognitive Sciences, Psychology, Education and Cultural Studies, University of Messina, Messina, Italy; ^2^Child and Adolescent Neurology and Psychiatric Section, Department of Clinical and Experimental Medicine, Catania University, Catania, Italy

**Keywords:** pediatric autoimmune neuropsychiatric disorders associated with streptococcal infections (PANDAS), pediatric acute-onset neuropsychiatric syndrome (PANS), autoimmune, neuropsychiatric disorders, streptococcal infection, diagnostic criteria

## Abstract

Pediatric autoimmune neuropsychiatric disorders associated with streptococcal infections (PANDAS) are clinical conditions characterized by the sudden onset of obsessive–compulsive disorder and/or tics, often accompanied by other behavioral symptoms in a group of children with streptococcal infection. PANDAS-related disorders, including pediatric acute-onset neuropsychiatric syndrome (PANS), childhood acute neuropsychiatric symptoms (CANS), and pediatric infection triggered autoimmune neuropsychiatric disorders (PITANDs), have also been described. Since first defined in 1998, PANDAS has been considered a controversial diagnosis. A comprehensive review of the literature was performed on PubMed and Scopus databases, searching for diagnostic criteria and diagnostic procedures of PANDAS and related disorders. We propose a test panel to support clinicians in the workout of PANDAS/PANS patients establishing an appropriate treatment. However, further studies are needed to improve our knowledge on these acute-onset neuropsychiatric conditions.

## Introduction

Pediatric autoimmune neuropsychiatric disorders associated with streptococcal infection (PANDAS) was first described by Swedo et al. in a group of 50 patients with an acute, sudden onset of obsessive–compulsive disorder (OCD) and/or tics, often accompanied by attention deficit/hyperactivity, separation anxiety, oppositional behaviors, and emotional lability, in the context of a previous streptococcal infection (1). Five working criteria for the diagnosis of PANDAS (Table 1) were proposed:

1. Presence of diagnostic criteria for OCD and/or tic disorder;
2. Pediatric onset, between 3 years and the beginning of puberty;
3. Episodic course of symptom severity, characterized by acute, severe onset and dramatic symptom exacerbations;
4. Temporal relationship between symptom onset and/or exacerbation and group A b-hemolytic streptococcal infections (GABHS); and
5. Association with neurologic abnormalities, such as motor hyperactivity, tics or choreiform movements.

**Table 1:** Diagnostic criteria of PANDAS, PANS, CANS, and PITANDs.

	**PANDAS**	**PANS**	**CANS**	**PITANDs**
OCD	++	++	++	++
Tic disorder	++	+	+	++
Restricted food intake	+/–	++	+/–	+/–
Neurological abnormalities	+	+	+	+
Relationship between symptoms and an infection	++	+/–	+/–	++
Anxiety	+/–	+	+	+/–
Emotional lability	+/–	+	+	+/–
Somatic symptoms	+/–	+	+	+/–
Behavioral regression	+/–	+	+	+/–
Deterioration in school performance	+/–	+	+/–	+/–
Behavioral abnormalities	+	+	+	+
Psychosis	+/–	+/–	+	+/–
Dysgraphia	+/–	+/–	+	+/–
Clumsiness	+/–	+/–	+	+/–
Hyperactivity	+/–	+/–	+	+/–

*The symbol +/− indicates the possible presence (+) or absence (−) of diagnostic features*.

Personality changes, cognitive disturbances, motor abnormalities, sensory sensitivity, behavioral regression, and, occasionally, psychosis are frequently observed (2, 3). The pathogenesis of PANDAS syndrome is hypothesized to be related to GABHS infections *via* molecular mimicry, where antibodies produced against streptococcal proteins also may cross-react with brain proteins, particularly in the basal ganglia (4). PANDAS exhibits immunological similarities to Sydenham's chorea (SC), a classic infection-triggered autoimmune disorder that also presents with a high degree of OCD comorbidity (5). Unlike SC, PANDAS has been considered a controversial diagnosis, because of contradictory results of various immunologic and epidemiologic studies, and the absence of clinical characteristics and biomarkers that differentiate PANDAS from childhood-onset OCD or tic disorders (6–8).

Swedo et al. (9) proposed a modification of the PANDAS diagnostic criteria termed “pediatric acute-onset neuropsychiatric syndrome (PANS).”

PANS is characterized by an abrupt onset of OCD and/or severe eating restrictions along with at least other concomitant cognitive, motor, behavioral, or affective symptoms (9). PANS criteria (Table 1) describe a clinically distinct presentation, defined as follows:

1. Abrupt, dramatic onset of OCD or severely restricted food intake (<48 h).
2. Concurrent presence of additional neuropsychiatric symptoms, with similarly severe and acute onset, from at least two of the following categories:
  A. Anxiety;
  B. Emotional lability and/or depression;
  C. Irritability, aggression, and/or severely oppositional behaviors;
  D. Behavioral (developmental) regression;
  E. Deterioration in school performance;
  F. Sensory or motor abnormalities including heightened sensitivity to sensory stimuli, hallucinations, dysgraphia, complex motor, and/or vocal tics; and
  G. Somatic signs or symptoms, including sleep disturbances, enuresis, or urinary frequency.
3. Symptoms are not better explained by a known neurologic or medical disorder, such as Sydenham chorea, systemic lupus erythematosus, Tourette syndrome, or others.

PANS symptoms overlap with a variety of psychiatric disorders, such as OCD, Tourette's syndrome, ADHD, depression, and bipolar disorder. Many clinical features are also shared between PANDAS and PANS. However, certain aspects of these two syndromes remain elusive, and there is a lack of a global agreement on their symptomatology and course. It is important to specify that PANDAS and PANS cannot occur in comorbidities. Furthermore, a streptococcal infection is required to make a diagnosis of PANDAS, but not for PANS.

Another PANDAS-related disorder but with a non-streptococcal infectious trigger was termed Pediatric Infection Triggered Autoimmune Neuropsychiatric Disorders (PITANDs). This acronym was coined to describe a subset of children with OCD who had a sudden onset of their psychiatric symptoms, typically following infection with a variety of agents (Varicella, *Mycoplasma pneumoniae*, etc.) (10) (Table 1). At this time, however, PITANDS diagnosis is no longer used.

Another group of authors, based on conflicting scientific support for the PANDAS syndrome, proposed the broader term “childhood acute neuropsychiatric syndrome” (CANS) for patients with acute, fulminant childhood onset of PANDAS symptoms but with no specificity regarding infections or autoimmunity (11) (Table 1). Ultimately, CANS diagnosis may be used in patients who meet the diagnostic criteria of PANDAS or PANS.

In sum, all these clinical conditions are characterized by a possible autoimmune etiology; however, the diagnosis is essentially based on the typical clinical presentation.

The aim of this study was (1) to collect and review the available data about the diagnostic evaluation of PANDAS and related disorders and (2) to focus on diagnostic accuracy of available investigations for diagnosis of these pediatric clinical conditions.

## Materials and Methods

### Search Strategy

A review of the PubMed and Scopus electronic databases was conducted on February 2021 for the following key terms: “Pediatric autoimmune neuropsychiatric disorders associated with streptococcal infections,” “Pediatric acute-onset neuropsychiatric syndrome,” “PANDAS,” “PANS” combined with “diagnosis,” “laboratory tests,” “diagnostic criteria,” to capture a broad range of potential articles. We did not restrain the period of search and reviewed all results. We included clinical studies with full-text display in English, which investigated the available diagnostic tools of these conditions, relevant for the clinician to these searches. Reference lists of all included papers were also checked for other relevant papers to the topic under review. Exclusion criteria were represented by studies written in other languages than English, letters to the editor, editorials, comments, animal studies, adult-focused studies, and contents not related to the topic of our review. At first, reviews, systematic reviews, textbooks, case reports, and case series were examined for any further publications but later excluded to this search.

### Data Extraction and Synthesis

A total of 783 articles were found through the databases. Data were searched and extracted independently by one author with assistance of the others. Of the 783 papers originally founded, 727 articles were ruled out based on exclusion criteria, and 56 were identified as potentially pertinent and included in the review.

## Results

### Laboratory Tests

There is currently no biomarker for the establishment of the diagnosis of these pediatric conditions. However, all patients meeting diagnostic criteria for PANDAS or PANS should have a series of laboratory tests: complete blood cell count with manual differential, indicator for liver and kidney disease, inflammatory markers such us erythrocyte sedimentation rate (ESR) and C-reactive protein (CRP), metabolic panel, and urinalysis (12, 13). The diagnosis of PANDAS is based on evidence of recent or current streptococcal infection, which is confirmed with the positive swab from the throat and/or increased titers of antistreptolysin-O (ASO) or anti-DNase B. Several studies showed simultaneously increased levels of ASO and anti-DNAse B in patients with PANDAS compared to controls (3, 14–20) (Table 2). However, the positive results do not differentiate between the carrier state and acute infection, they only indicate exposure to the streptococcal infection (13). Furthermore, false positive or false negative of either ASO or anti-DNase B results is possible. In case of suspected diagnosis of PANS, other infectious triggers should be considered. *Mycoplasma pneumoniae*, viruses including influenza and Epstein–Barr virus, and *Borrelia burgdorferi* (Lyme disease) have also been reported as a potential infectious trigger and should be included in the infectious agent's evaluation for PANS (12, 24).

**Table 2:** Studies that investigated titers of antistreptolysin-O (ASO) and anti-DNase B in patients with PANDAS.

**References**	**No. of patients/controls**	**ASO**	**Anti-DNAse B**
Pavone et al. (14)	22/22	> control	> control
Luo et al. (21)	47/19	= control	= control
Kurlan et al. (15)	40/40	> control	> control
Morris et al. (16)	30/30	> control	> control
Bernstein et al. (2)	21/19	= control	= control
Murphy et al. (3)	41/68	> control	> control
Morris-Berry et al. (22)	44/24	= control	= control
Murphy et al. (23)	43/69	= control	= control
Stagi et al. (17)	77/191	> control	> control
Cox et al. (18)	311/16	> control	> control
Stagi et al. (19)	179/224	> control	> control
Chain et al. (20)	60/28	> control	> control

Additional laboratory investigations were performed in clinical studies regarding PANDAS disease. D8/D17, a monoclonal antibody directed against a B lymphocyte surface antigen, has been proposed as a potential marker of susceptibility to PANDAS (21, 25, 26) (Table 3). However, D8/D17 is no longer used owing to poor specificity for PANDAS diagnosis (37–39). Given its role in the modulation of innate immunity and autoimmunity, vitamin D levels have been investigated in several studies, with detection of low levels in PANDAS (19, 27) as well as in PANS patients (28–30) (Table 3). Evidence from a recent study also suggested that lower vitamin D levels may be associated with the presence and severity of comorbid ADHD in children and adolescents with chronic tic disorders (40). Low vitamin D levels may occur in various neurodevelopmental disorders such as autism spectrum disorder (41), making unlikely the usefulness of Vitamin D dosage as potential biomarker of PANDAS condition. Recent studies have also evaluated ferritin and iron levels in patients with PANS. Hypoferritinemia was documented in two different pediatric PANS cohorts (29, 31), associated in one case with low serum iron levels (31) (Table 3). Abnormalities of thyroid function and thyroid autoimmune diseases, as well as the association with celiac disease, have been explored in a few studies (17, 28–30, 32). Elevated transglutaminase and antithyroid antibodies have been documented in, respectively, 1.2–11% and 2.46–36% of patients affected with PANDAS or PANS, whereas TSH abnormalities and/or low T4 have been found in 3.8–11% (17, 28–30, 32) (Table 3). Moreover, evaluation for autoimmune disease through measurements of serum antinuclear antibody (ANA) or others (b2-glycoprotein antibodies, anticardiolipin antibodies, antiphospholipid antibody, and lupus anticoagulant) has yielded conflicting results. Positive ANA titers have been found in 0.5–37.1% of PANDAS/PANS patients (17, 20, 28–30, 32–35), while no other autoantibodies were detected (17, 35) (Table 3). Furthermore, Murphy et al. (3, 23) have documented elevated anti-A carbohydrate (anti-A_CHO_) antibody levels in PANDAS patients compared to healthy controls, while Frankovich et al. (28) have reported positive histone antibodies in 15% of PANS patients (Table 3). Finally, other studies concerning immune parameters (Ig subclasses and cytokine) have been shown altered immunoglobulin profiles and cytokine levels in PANS patients (IL1β, IL6, TNFα, IL8, and IL10) (28–30, 34, 36) (Table 3). Regarding these additional diagnostic tests, it should be specified that none of these biomarkers are specific for the diagnosis, but nevertheless should be examined for the differential diagnosis.

**Table 3:** Studies about various laboratory tests in patients with PANDAS or PANS.

**Laboratory tests**	**References**	**Results**
Vitamin D	Celik et al. (27), Stagi et al. (19), Frankovich et al. (28), Gromark et al. (29), and Gromark et al. (30)	Low levels both in PANDAS than in PANS patients.
Ferritin	Gromark et al. (29) and Chan et al. (31)	Hypoferritinemia in PANS patients.
Iron	Chan et al. (31)	Low serum iron levels in PANS patients.
D8/17-reactive cells	Swedo et al. (25), Luo et al. (21), and Sokol et al. (26)	High levels in PANDAS patients, but poor specificity.
Celiac test	Stagi et al. (17), Frankovich et al. (28), Gromark et al. (29), and Gromark et al. (30)	Elevated transglutaminase antibodies in 1.2%−11% of patients with PANDAS/PANS.
Thyroid tests	Stagi et al. (17), Frankovich et al. (28), Gromark et al. (29), Gagliano et al. (32), and Gromark et al. (30)	Elevated antithyroid antibodies in 2.46%−36%, and TSH abnormalities and/or low T4 in 3.8%−11% of patients with PANDAS/PANS.
Antinuclear antibody (ANA)	Chain et al. (20), Stagi et al. (17), Williams et al. (33), Gromark et al. (29), Murphy et al. (34), Gagliano et al. (32), Lepri et al. (35), Gromark et al. (30), and Frankovich et al. (28)	Positive ANA titers in 0.5%−37.1% of PANDAS/PANS patients.
Anti-A carbohydrate (anti-ACHO) antibody	Murphy et al. (3, 23)	Elevated levels in PANDAS patients.
Histone antibodies	Frankovich et al. (28)	Positive titers in 15% of PANS patients.
Immune parameters (Ig subclasses, cytokine)	Frankovich et al. (28), Murphy et al. (34), Murphy et al. (36), Gromark et al. (29), and Gromark et al. (30)	Altered immunoglobulin profiles and cytokine levels (IL1β, IL6, TNFα, and IL8, IL10) in PANS patients.

### Neural Autoimmunity

Based on the evidence for autoantibodies in SC, it is hypothesized that the underlying pathology in PANDAS involves an immune-mediated mechanism with molecular mimicry. Therefore, several studies have investigated the role of cross-reactive antibodies in patients with PANDAS and related disorders. Accumulating evidence with controversial results showed that autoantibodies target antigens in the basal ganglia (14, 16, 22, 38, 42–50). Autoimmune targets identified to overlap between SC and PANDAS were used to develop the Cunningham Panel, a commercially available set of blood tests utilized for measuring immune dysfunction related to neuropsychiatric conditions associated with an infection trigger (18, 51, 52). This panel aims to titer autoantibodies to dopamine receptors D1 and D2, tubulin and lysoganglioside-GM1, and calcium/calmodulin-dependent protein kinase II (CaMKII). Despite the fact that multiple studies have analyzed biomarker levels in patient populations and comparison samples (18, 22, 33, 45–49, 53), only a few studies have measured all five assays of this panel of antibodies simultaneously (18, 20, 50, 52, 54). Cox et al. (18) suggested a significant correlation of streptococcal-associated tics and OCD with elevated anti-D1R and antilysoganglioside antineuronal antibodies in serum concomitant with higher activation of CaMKII in human neuronal cells. In another study performed in children with PANDAS-chronic tics and OCD (50), CaMKII activation at the GABHS exacerbation was identified in some subjects. While the clinical use of the Cunningham Panel in diagnosing PANS or PANDAS is not supported by Hesselmark and Bejerot (54), results reported by other recent studies suggested the clinical utility of the Cunningham Panel (20, 52). Despite multiple studies, the clinical reliability of these autoantibodies remains unclear, given the various and often poorly reproducible techniques used for their analysis. Finally, based on the results of a small pilot study (55), Xu et al. (56) investigated the cellular effects of antibodies on cholinergic interneurons (CINs) from 27 children with PANDAS and 23 control subjects. PANDAS serum IgG showed selectively elevated binding to CINs of the striatum, offering novel evidence for striatal CINs as a critical cellular target for antibodies in these patients (56). However, future research is needed to improve our knowledge about these molecular targets of pathogenic antibodies in PANDAS patients.

### Lumbar Puncture

Lumbar puncture (LP) with a complete cerebrospinal fluid (CSF) evaluation and assays for antineuronal antibodies should be considered if there are MRI or electroencephalographic (EEG) abnormalities, or encephalopathic symptoms (12, 13). Diagnostic criteria for possible autoimmune encephalitis include a subacute onset of working memory deficits, altered mental status or psychiatric symptoms associated at least to one of the following: (1) new focal CNS findings, (2) seizures not explained by a previously known seizure disorder, (3) CSF pleocytosis, and (4) MRI features suggestive of encephalitis. Clearly, other possible alternative causes must be ruled out (57). There is a heterogeneous presentation of neuropsychiatric features of autoimmune encephalitis in the pediatric population. Symptoms of autoimmune encephalitis can include cognitive regression/impairment, memory changes, seizures, sleep disturbance, autonomic instability, speech changes or mutism, and involuntary movement (58). LP may be crucial for a correct differential diagnosis.

### Electroencephalography and Others Electrophysiological Investigations

Although sleep disturbances are frequently reported in PANDAS or PANS patients, little data are currently available in literature regarding electrophysiological investigations in these patients. EEGs may be a useful tool in demonstrating possible signs of abnormal brain activity. Intermittent or persistent focal or generalized EEG alterations were reported in 22 patients (56%) affected by PANS (32). Focal epileptiform activity was reported by Gamucci et al. (59) in a pediatric PANDAS and PANS sample, but EEG monitoring was not available for all participants. Similarly, polysomnography (PSG) evaluations showed several sleep disruptions in children with PANS, such as rapid eye movement (REM) sleep motor disinhibition (60) or periodic limb movement during REM sleep (61). However, there are few data regarding the application of electrophysiological investigations in this population. Further studies into the sleep disturbances of patients with PANS are needed.

### Neuroimaging

Neuroimaging studies on pediatric patients affected by PANDAS or PANS are limited. After the demonstration of a reduction of magnetic resonance images (MRI) and basal ganglia volumes after plasmapheresis in a single case study of an adolescent with PANDAS (62), the cerebral MRI of 34 children with PANDAS were compared to those from 82 healthy controls. The average size of the caudate, putamen, and globus pallidus, but not of the thalamus or total cerebrum, were significantly greater in the PANDAS group than the control (63) (Table 4). Furthermore, volumetric increases in basal ganglia structures found among the children with PANDAS were like those reported for Sydenham's chorea (SC) (67). The neuroimaging studies are consistent with the hypothesis of a selective cross-reactive antibody-mediated inflammation of the basal ganglia underlying the development of post-streptococcal OCD or tics (63). Using voxel-based morphometry, diffusion tensor imaging (DTI), and surface analysis, Cabrera et al. (65) documented neuroanatomical differences for the gray and white matter pattern between subjects with PANDAS and healthy controls, but no significant group differences in DTI measures or cortical features (65) (Table 4). Other neuroimaging studies on PANDAS or PANS patients showed no brain MRI anomalies (59, 64) or described the detection of minor structural “incidental” abnormalities not located in the basal ganglia or cortical areas (32) (Table 4). A recent case–control study of 34 patients with PANS demonstrated increased diffusion patterns in all assessed brain regions, particularly the deep gray matter (thalamus, basal ganglia, and amygdala). These cerebral microstructural differences are consistent with the cardinal clinical symptoms of these patients, suggesting a unifying inflammatory process involving the central nervous system, particularly the basal ganglia (66) (Table 4). Finally, by using positron emission tomography (PET) imaging with (11)C-[R]-PK11195 (PK), a ligand that binds to the transporter protein expressed by activated microglia, Kumar et al. (64) presented increased PK binding potential values, suggesting underlying neuroinflammation, in bilateral caudate for both PANDAS and Tourette syndrome (TS) patients, and in bilateral lentiform nuclei in patients with PANDAS only (Table 4).

**Table 4:** Neuroimaging studies in patients with PANDAS or PANS.

**References**	**N^°^ patients**	**Results**
Giedd et al. (63)	34 PANDAS	**MRI**: The average size of the caudate, putamen, and globus pallidus, but not the thalamus, were larger in the PANDAS group than the control.
Kumar et al. (64)	29 (17 PANDAS, 12 TS)	**MRI**: Normal. **PET scanning**: Activated microglia-mediated neuroinflammation, increased in bilateral caudate and bilateral lentiform nucleus in the PANDAS group and in bilateral caudate nuclei only in the TS group, compared to control group.
Cabrera et al. (65)	14 PANDAS	**MRI**: The pattern for the gray and white matter was significantly different between subjects with PANDAS and controls. Alterations emerged in the cortex, subcortex, and cerebellum. There were no significant group differences in DTI measures or cortical features.
Gamucci et al. (59)	11 (6 PANS, 5 PANDAS)	**MRI**: Normal.
Gagliano et al. (32)	39 PANS	**MRI:** Minor structural ≪incidental≫ abnormalities (no specific volumetric and/or inflammatory changes in the basal ganglia or cortical areas) in 21% of the sample.
Zheng et al. (66)	34 PANS	**MRI**: Increased diffusion patterns in all assessed brain regions, particularly the deep gray matter (thalamus, basal ganglia, and amygdala), compared with control participants.

### Other Diagnostic Evaluations

PANDAS syndrome is characterized by several clinical features that may involve multiple districts, but there are only a few data in the literature about these aspects. Considering the detection of significant echocardiographic findings in patients with rheumatic fever and SC, the cardiologic involvement in the PANDAS subgroup was first investigated by Snider et al. (68). Children with PANDAS were assessed by echocardiography, and only one patient (1.7%) was found to have mild mitral regurgitation (68) (Table 5). In contrast, full cardiological assessment was performed through clinical examination, electrocardiography (ECG), and echocardiography in a selected pediatric population diagnosed with PANS/PANDAS (69). A significant number of children presented systolic murmurs (60%), ECG abnormalities (6.6%), and mild mitral valve involvement (20%), suggesting that a cardiologic screening should be performed in these patients (69) (Table 5).

**Table 5:** Other diagnostic evaluations in patients with PANDAS or PANS.

**References**	**No. of patients**	**Results**
Snider et al. (68)	60 PANDAS	Mild mitral regurgitation (1.7%).
Murciano et al. (69)	30 PANS/PANDAS	Cardiac auscultation abnormalities (60%). ECG abnormalities (6.6%). Echocardiography abnormalities (20%).
Quagliarello et al. (70)	30 PANS/PANDAS	Alterations of gut microbial biomarkers.
Loffredo et al. (71)	30 PANDAS	Increased serum levels of oxidative stress and gut-derived lipopolysaccharides (LPS).
Cocuzza et al. (72)	130 PANDAS	Otorhinolaryngologic symptoms (67.7%).
Luleyap et al. (73)	42 PANDAS	TNF-α gene promoter region−308 G/A AA polymorphism in 32/42 (76.2%).
Celik et al. (74)	59 PANDAS, 23 PANDAS variants	The presence of any variant of MBL2 gene was found in 14.50-fold increased frequency in the PANDAS subgroup compared with the non-PANDAS subgroup.

Recently, there has been a growing interest in alterations of gut microbiota (GM), particularly regarding its possible correlations with neuroinflammation and the development of psychiatric disorders. Quagliarello et al. (70) investigated the gut microbiota profiling in PANDAS patients, suggesting that alterations of gut microbial biomarkers can lead to a proinflammatory state (Table 5). A recent study performed by Loffredo et al. (71) also shows that children affected by PANDAS have high circulating levels of soluble NOX2-dp (sNOX-2-dp), isoprostanes, and lipopolysaccharide (LPS), as markers of oxidative stress that could be involved in the process of neuroinflammation (Table 5). Overall, these observations may lead to the conclusion that alterations in the gut microbiota may represent one of the factors enhancing PANDAS development (75).

Otorhinolaryngology symptoms (ENT) in a pediatric population affected by PANDAS were found in about 67.7% of patients, suggesting that PANDAS patients may undergo a specific otolaryngologic consultation (72) (Table 5). Finally, genetic investigations in patients with PANDAS showed a positive relationship with the TNF-α gene promoter region−308 G/A AA polymorphism (73) and exon variants of the MBL2 gene (Mannose-binding lectin) (74) (Table 5).

### Neuropsychological Assessment

Neurocognitive functioning of patients affected by PANDAS and related disorders has not been extensively studied in literature. Lewin et al. (76) evaluated neurocognitive profiles in 26 youth with PANDAS, and observed severe impairment in visuospatial recall memory, inhibitory control, fine motor speed, and graphomotor function. Reduced sustained attention and response suppression have also been reported in patients with PANDAS (77). Bernstein et al. (2) compared clinical characteristics of children with PANDAS and children with non-PANDAS OCD. There was no significative difference between the two-diagnostic group on OCD severity score, while the tic severity score was significantly higher in the PANDAS group compared with the non-PANDAS group (2). A recent study has also demonstrated relative difficulties with aspects of executive functions and motor skills in patients with PANDAS (78). Instead, the neuropsychological profile of PANS subjects was analyzed in a few studies. Murphy et al. (34) demonstrated visuospatial deficit in a cohort of 43 youth with PANS. A study of cognitive functioning in patients with PANDAS and related disorders has found a more compromised neuropsychological profile in patients with PANS with respect to patients with PANDAS, particularly for visual-motor abilities, short- and long-term memory, and processing speed (59). In some studies (34, 79–81), PANS patients have been assessed using PANS scales, in association to other self-report and clinician administered measures for a comprehensive clinical evaluation. Furthermore, the use of specific PANS-related scales do not seem to have added value to clarify the diagnostics of Tourette syndrome [European Clinical guidelines for Tourette syndrome and other tic disorders Part 1 Assessment-2,0 (82)].

## Discussion

Although there is a body of evidence that supports the existence of PANDAS and related conditions, it remains a controversial diagnosis (83). However, some authors who have investigated the PANDAS hypothesis do support the existence of these conditions (16, 22, 42, 44, 46–50, 53, 54, 84, 85). More studies failed to identify significant differences in specific serum autoantibodies between PANDAS patients and healthy controls (16, 42, 46, 47). Until now, none of the studied diagnostic approaches is sufficient to confirm the diagnosis. The responsibility of evaluating these patients falls to primary care clinicians and child psychiatrists (12). As shown, the diagnosis for the PANDAS syndrome and related conditions is clinical and it requires a complete medical and psychiatric history, a thorough physical examination, a comprehensive neuropsychological assessment, and a series of lab tests. More specific blood tests should also be performed in case of suspected alternative medical explanations for the neuropsychiatric symptoms. If PANS is suspected, it is recommended to consider other possible infectious agents such us *Mycoplasma pneumoniae*, EBV, and *Borrelia burgdorferi*. Two unrelated cases of PANS onset in patients with a COVID-19 infection have lately been reported (86). Furthermore, a recent study explored the effects of the COVID-19 pandemic during the lockdown on children affected by PANS and showed an increase in symptoms during the block in 71% of the sample (87). Therefore, COVID-19 infection should also be considered in the differential diagnosis. It is important to underline that various research groups have reported different results about lab tests, due to the use of different analytical techniques.

Based on present data, the diagnostic evaluation of children who meet PANDAS or PANS diagnostic criteria would benefit from including a cardiologic screening with ECG and EEG examination. Given its poor specificity, the clinical use of the Cunningham Panel in diagnosing PANS or PANDAS is not supported for patients with mild forms of the disease. Instead, antineuronal antibodies in serum and CSF, and MRI are indicated for the differential diagnosis with autoimmune encephalitis (7, 12). Based on the results obtained from the previously performed diagnostic investigations, it may be necessary to carry out further paraclinical investigations such as echocardiography or PSG.

By definition, PANDAS and related disorders are always a diagnosis of exclusion. Differential diagnosis is performed with other similar clinical conditions and requires a multidisciplinary approach. Given these diagnostic difficulties, there is a clear need to a better definition of PANDAS/PANS syndrome, to establish more precise diagnostic guidelines and indications. With respect to the previously published PANS diagnostic guidelines (12) and other proposed diagnostic protocols (59, 88), we propose a test panel to support clinicians in the management of PANDAS/PANS patients to promptly initiate a treatment when appropriate (Supplementary Figure 1).

The present scheme may also be helpful in the differential diagnosis with other childhood-onset neuropsychiatric disorders (i.e., autoimmune acute encephalitis) to define their diagnosis as soon as possible. In order to understand which of the available diagnostic tests are most discriminating for the PANDAS/PANS diagnosis, more systematic data should be collected, which can later be used to set the most appropriate diagnostic protocol(s). In conclusion, larger studies are desirable, to establish more precise diagnostic guidelines and indications.

## Author Contributions

AP and MS drafted the manuscript. MG, RB, CV, and RR performed critical editing. AP, RR, and CV participated in constructive outline, discussions, and editing. All authors read and approved the final manuscript.

## Conflict of Interest

The authors declare that the research was conducted in the absence of any commercial or financial relationships that could be construed as a potential conflict of interest.

## Publisher's Note

All claims expressed in this article are solely those of the authors and do not necessarily represent those of their affiliated organizations, or those of the publisher, the editors and the reviewers. Any product that may be evaluated in this article, or claim that may be made by its manufacturer, is not guaranteed or endorsed by the publisher.

## Supplementary Material

Supplementary Figure 1 Proposed test panel for patients with suspected PANDAS and related disorders.
