# Supplementary material for: Diagnostic Approach to Pediatric Autoimmune Neuropsychiatric Disorders Associated With Streptococcal Infections (PANDAS): A Narrative Review of Literature Data
Source: Front Pediatr. 2021 Oct 27;9:746639. doi: 10.3389/fped.2021.746639 (PMC8580040; doi:10.3389/fped.2021.746639)
Supplement: Supplementary Figure 1 — Proposed test panel for patients with suspected PANDAS and related disorders. [file Data_Sheet_1.PDF]

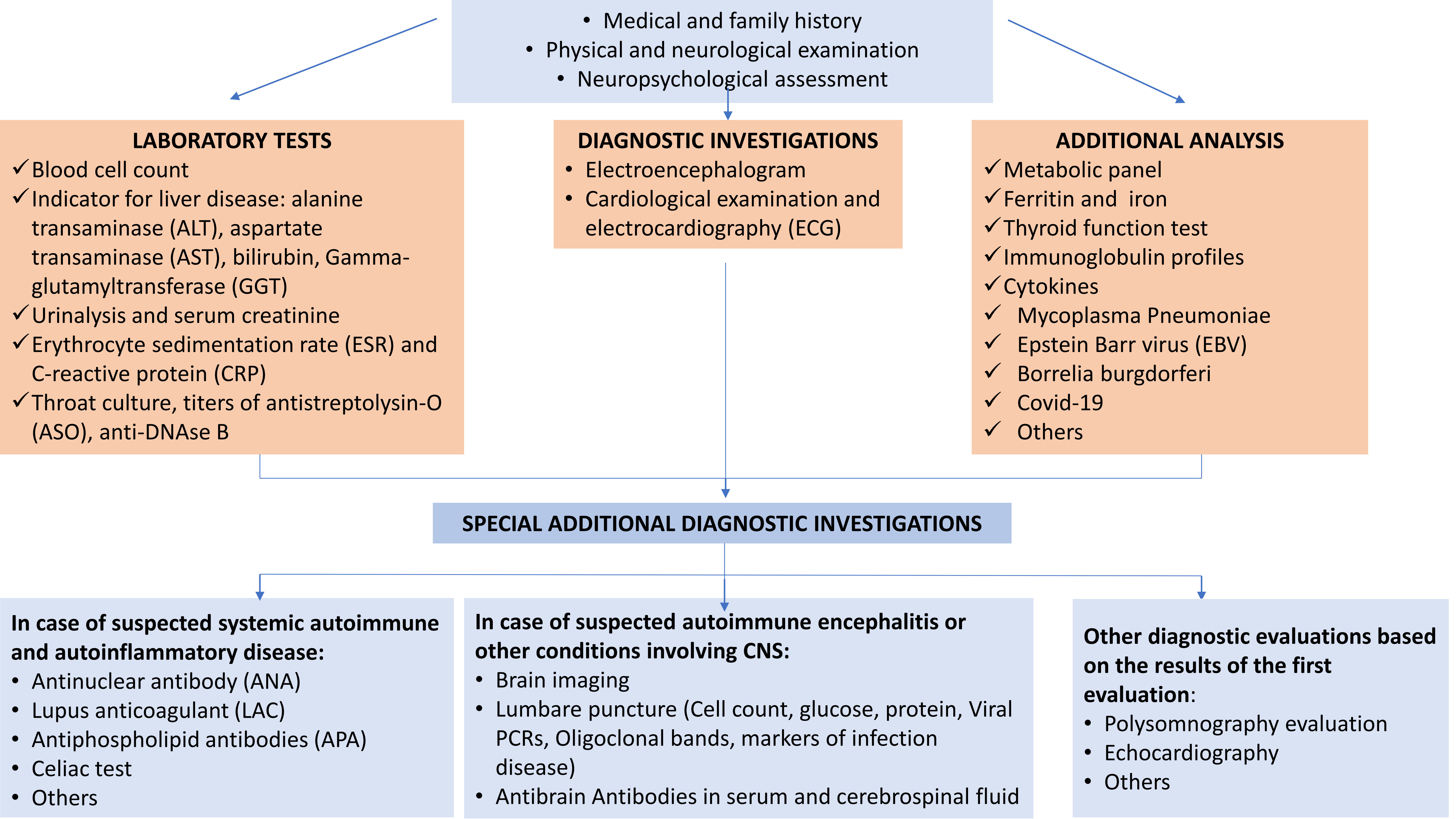

• Medical and family history
• Physical and neurological examination
• Neuropsychological assessment
LABORATORY TESTS
✓Blood cell count
✓Indicator for liver disease: alanine transaminase (ALT), aspartate transaminase (AST), bilirubin, Gamma-glutamyltransferase (GGT)
✓Urinalysis and serum creatinine
✓Erythrocyte sedimentation rate (ESR) and C-reactive protein (CRP)
✓Throat culture, titers of antistreptolysin-O (ASO), anti-DNAse B
DIAGNOSTIC INVESTIGATIONS
• Electroencephalogram
• Cardiological examination and electrocardiography (ECG)
ADDITIONAL ANALYSIS
✓Metabolic panel
✓Ferritin and iron
✓Thyroid function test
✓Immunoglobulin profiles
✓Cytokines
✓ Mycoplasma Pneumoniae
✓ Epstein Barr virus (EBV)
✓ Borrelia burgdorferi
✓ Covid-19
✓ Others
SPECIAL ADDITIONAL DIAGNOSTIC INVESTIGATIONS
In case of suspected systemic autoimmune and autoinflammatory disease:
• Antinuclear antibody (ANA)
• Lupus anticoagulant (LAC)
• Antiphospholipid antibodies (APA)
• Celiac test
• Others
In case of suspected autoimmune encephalitis or other conditions involving CNS:
• Brain imaging
• Lumbare puncture (Cell count, glucose, protein, Viral PCRs, Oligoclonal bands, markers of infection disease)
• Antibrain Antibodies in serum and cerebrospinal fluid
Other diagnostic evaluations based on the results of the first evaluation:
• Polysomnography evaluation
• Echocardiography
• Others
